# Predictors of Responsiveness to GLP-1 Receptor Agonists in Insulin-Treated Patients with Type 2 Diabetes

**DOI:** 10.1155/2023/9972132

**Published:** 2023-08-08

**Authors:** Colleen Gavigan, Thomas Donner

**Affiliations:** Johns Hopkins Diabetes Center, Division of Endocrinology, Diabetes & Metabolism, Johns Hopkins University School of Medicine, 601 N Caroline Street, Baltimore, Maryland, USA 21287

## Abstract

**Background:**

Glucagon-like peptide-1 receptor agonists (GLP-1 RAs) are potent antihyperglycemic agents with beneficial effects on weight, cardiovascular, and renal outcomes. Physicians lack guidance as to which patients with insulin-requiring type 2 diabetes will respond best to GLP-1 RAs with respect to glycemic control, insulin dose reduction, and weight loss. This study evaluated the efficacy of GLP-1 RAs in patients with type 2 diabetes on insulin and patient factors that may predict a beneficial clinical response.

**Methods:**

Adults with type 2 diabetes treated with insulin who had a GLP-1 RA added to their regimen were evaluated retrospectively. Baseline parameters and outcomes at 3, 6, and 12 months were collected.

**Results:**

Among the 81 patients included, there was a mean reduction in hemoglobin A1C of 0.94% (SD, 0.26; *p* = 0.0007), 0.40% (SD, 0.21; *p* = 0.0636), and 0.58% (SD, 0.23, *p* = 0.0154) at 3, 6, and 12 months, respectively, following the addition of a GLP-1 RA. There was also a reduction in body weight noted at each time point. Baseline characteristics including BMI, duration of diabetes, and insulin requirement did not significantly affect A1C reduction when GLP-1 RA was added. At 3 months, patients with a random C-peptide that was normal (≥0.8 ng/ml) were significantly more likely to have discontinued insulin than those with random C-peptide that was low (<0.8 ng/ml) (11 of 23 vs. 0 of 7 patients, *p* = 0.029).

**Conclusions:**

The addition of a GLP-1 RA reduced HbA1C, weight, and insulin requirements in this cohort of patients with type 2 diabetes on insulin. BMI, baseline insulin dose, and diabetes duration did not predict response. A C-peptide level ≥ 0.8 ng/ml predicted a beneficial response after 3 months of therapy.

## 1. Introduction

Patients with uncontrolled type 2 diabetes are commonly treated with long- and rapid-acting insulins, the most effective class of antihyperglycemic agents available. Insulin use is associated with an increased risk of hypoglycemia and weight gain [1, 2]. Interventions that reduce the need for insulin in patients with diabetes may be associated with long-term beneficial outcomes.

Glucagon-like peptide-1 receptor agonists (GLP-1 RAs) are potent antihyperglycemic agents that function via glucose-dependent insulin secretion, as well as inhibition of appetite, gastric emptying, and glucagon secretion [3]. These agents have additionally been associated with weight loss, a reduction in major adverse cardiovascular events, improvement in fatty liver, and have renal protective effects [4–7]. The addition of a GLP-1 RA to basal insulin instead of using traditional prandial insulin is now increasingly chosen based on similar hemoglobin A1C (HbA1C) reduction and the additional benefits of weight loss and reduced risk of hypoglycemia [8]. In patients with type 2 diabetes on multidose insulin injections (MDI), randomization to treatment with the GLP-1 RA albiglutide reduced the number of prandial insulin injections, improved glycemic control, led to weight loss, and resulted in less hypoglycemia when compared with continuation of MDI without albiglutide [9]. As a result of these and similar findings, GLP-1 RAs are more commonly being added to the regimen of patients taking basal or basal-bolus insulin.

Few studies to date have evaluated predictors of response to the addition of a GLP-1 RA in patients already on insulin therapy [10–13]. There remains a lack of clear guidance on which patient populations benefit most from the addition of these agents with respect to glycemic control, insulin dose reductions, and weight loss.

This study sought to confirm the prior findings of metabolic benefits when a GLP-1 RA is added to insulin therapy, and identify baseline patient characteristics including HbA1C, insulin dose, body mass index (BMI), and random C-peptide, that may act as predictors of response. C-peptide has been shown to be a useful test to assess the degree of residual endogenous insulin secretion in patients with type 1 and type 2 diabetes [14–16]. Patients with type 2 diabetes and reduced insulin secretion have previously been shown to respond less well to noninsulin antihyperglycemic agents [17–21].

## 2. Methods

This retrospective observational analysis was reviewed by the Johns Hopkins Medicine Institutional Review Board and deemed exempt research under DHHS regulations. A query of the electronic medical record generated 177 charts associated with insulin and GLP-1 RA. This was followed by manual chart review which identified 81 patients who met the following inclusion criteria: adults aged 18 and older with a diagnosis of type 2 diabetes; seen by the principal investigator at the Johns Hopkins Diabetes Center between 2010 and 2020; treated with insulin and subsequently had a GLP-1 RA (liraglutide, semaglutide, or dulaglutide) added to their insulin regimen. The initial EMR query was prone to error and required removal of the following patients identified as not meeting criteria on manual review: 2 patients were deceased, 31 patients were not on both insulin and a GLP-RA simultaneously, 4 patients were on exenatide, 10 patients stopped the GLP-1 RA due to adverse effects (none requiring hospitalization), 1 patient was pregnant, 5 patients were found to have type 1 diabetes, 7 never started treatment due to cost of GLP-1 RA, and 36 did not have adequate data or follow-up. Treatment was initiated with the starting dose of the GLP-1 RA chosen and progressively titrated up to the maximally well-tolerated dose; this progression varied, and sometimes patients were increased after many months if tolerability was limited initially. Baseline parameters were collected including age, sex, race/ethnicity, weight, BMI, duration of diabetes, random C-peptide level (when available), use of noninsulin agents, choice of GLP-1 RA, HbA1C, and dose of basal and prandial insulins. Outcome data at 3, 6, and 12 months following initiation of GLP-1 RA included weight, use of noninsulin agents, HbA1C, and dose of basal and prandial insulin. Patients were excluded if the GLP-1 RA was discontinued prior to 3 months.

A smaller population of 49 patients who met the above criteria and in whom a C-peptide level was available, was also evaluated with a more limited data set including baseline random C-peptide as well as HbA1C, weight, and basal and prandial insulin doses at baseline, 3, 6, and 12 months when available.

### 2.1. Statistical Methods

Paired *t*-tests were used to calculate change in weight and change in HbA1C with standard error at each time point and at different C-peptide cutoffs. Two-sided Fisher's exact tests were used to evaluate the number of patients who had stopped prandial insulin at different C-peptide cutoffs at each time point. A *p* value <0.05 was considered statistically significant.

## 3. Results

Baseline demographics and clinical characteristics of the 81 patients involved in primary analysis were as follows: mean age, 61.5 years (SD, 11.4); men, 51 (64%); White, 44 (55%); Black, 25 (31%); Asian, 2 (2.5%); Hispanic, 2 (2.5%); mean BMI, 34.4 kg/m^2^ (SD, 6.6); mean diabetes duration, 16.9 years (SD, 9.6); treated with metformin, 54 (67.5%); treated with other antihyperglycemics, 44 (54.3%); mean HbA1C, 8.2% (SD, 1.5); and mean total daily dose of insulin, 82.4 units (SD, 72.3). Of the patients evaluated, 18 (23%), 51 (64%), and 11 (14%) took dulaglutide, liraglutide, and semaglutide, respectively (Table 1).

Primary analysis demonstrated a mean reduction in HbA1C of 0.94% (SD, 0.26; *p* = 0.0007), 0.40% (SD, 0.21; *p* = 0.0636), and 0.58% (SD, 0.23, *p* = 0.0154) at 3, 6, and 12 months, respectively, with the addition of a GLP-1 RA. A mean reduction in weight of 4.90 kg (SD, 1.40; *p* = 0.001), 4.41 kg (SD, 0.79; <0.0001), and 5.64 kg (SD, 0.86 kg; *p* < 0.0001) was observed at 3, 6, and 12 months, respectively (Table 2).

Among the 47 patients on prandial insulin at baseline, 20 (42%) had discontinued it by 3 months, 14 (30%) by 6 months, and 12 (26%) by 12 months.

There was no significant difference in mean change in HbA1C at 3, 6, or 12 months (*p* values 0.99, 0.98, and 0.95, respectively) in patients with BMI <30 compared to those with BMI ≥ 30 kg/m^2^. There was no significant difference in percentage of patients who discontinued prandial insulin at 3, 6, or 12 months (*p* values 0.75, 0.13, and 0.72, respectively) in patients with BMI <30 compared to those ≥30 kg/m^2^.

The change in HbA1C at 3, 6, or 12 months (*p* values 0.99, 0.98, and 0.95, respectively) was not significantly different in patients with diabetes for <10 years compared to those with diabetes for ≥10 years. There was no significant difference in percentage of patients who discontinued prandial insulin at 3, 6, or 12 months (*p* values 0.75, 0.13, and 0.72, respectively) in patients with diabetes for <10 years compared to those with diabetes for ≥10 years.

There was no significant difference in mean change in HbA1C at 3, 6, or 12 months (*p* values 0.97, 0.26, and 0.78, respectively) in patients with baseline insulin requirement of <0.8 units/kg/day compared with those requiring ≥0.8 units/kg/day. There was no significant difference in percentage of patients who discontinued prandial insulin at 3, 6, or 12 months (*p* values 0.36, 0.09, and 0.19, respectively) in patients with a baseline insulin requirement of <0.8 units/kg/day compared with those requiring ≥0.8 units/kg/day.

In the cohort of 49 patients selected for having random C-peptide data available, when low C-peptide, defined as less than 0.8 ng/ml, was compared with normal/high C-peptide, defined as ≥0.8 ng/ml, more patients were able to discontinue prandial insulin in the normal/high C-peptide group than in the low C-peptide group. This was significant at 3 months (*p* = 0.029) (Table 3). The reduction in HbA1C was numerically greater in the normal/high C-peptide group than the low C-peptide group at 3 months (1.01% vs. 0.63%, respectively) and at 12 months (0.91% vs. 0.68%, respectively) but not statistically significant at either time point (*p* = 0.252 and *p* = 0.539 at 3 and 12 months, respectively) (Table 4). The mean C-peptide was higher in patients who stopped insulin vs. those who did not at 3 and 6 months (2.21 vs. 2.14 ng/ml and 2.59 vs. 1.60 ng/ml, respectively) but not statistically significant (*p* = 0.925 and *p* = 0.057 at 3 and 6 months, respectively). When evaluated as three groups (low, normal, and high C-peptide), more patients in the normal C-peptide range were able to stop prandial insulin compared with the other groups at all-time points, although this was not significant (*p* = 0.063, 0.090, and 0.316 at 3, 6, and 12 months, respectively). The *N* for each of these analyses varied due to missing data at some time points.

Among those patients on basal-bolus insulin (*N* = 38), the mean insulin dose reduction was 49.4 units (SD, 48.6), 84.3 units (SD, 70.3), and 52.0 units (SD, 18.0) at 3, 6, and 12 months, respectively, in patients who discontinued prandial insulin vs. 19.4 (SD, 31.7), 24.5 (SD, 46.6), and 30.9 units (SD, 58.1), respectively, in those patients who remained on prandial insulin for the 12-month duration. Given the wide variability in insulin dose requirements, the differences between these groups were not significant, although there was a trend of a larger dose reduction in those patients who discontinued prandial insulin (Table 5).

## 4. Discussion

In a selected population of individuals with type 2 diabetes on insulin therapy, this study confirmed that the addition of a GLP-1 RA (liraglutide, semaglutide, or dulaglutide) effectively reduced HbA1C, body weight, and insulin requirements. The addition of these agents also enabled a proportion of patients with type 2 diabetes on multiple daily injections to discontinue prandial insulin: 42% of patients on prandial insulin discontinued it by 3 months following GLP1 RA initiation. Similar findings were previously well demonstrated in a prospective randomized controlled trial by Rosenstock et al. in which the addition of a GLP-1 RA to a basal-bolus insulin regimen led to reduction in prandial insulin requirement. In fact, 54% of patients in that study discontinued prandial insulin entirely [9].

In our population of patients with type 2 diabetes on insulin, GLP-1 RAs appear to have similar efficacy in terms of A1C reduction and discontinuation of prandial insulin regardless of baseline BMI, duration of diabetes, or baseline total daily insulin dose when analyzed with specific cutoffs in these categories. Other studies that have looked at predictors of response to GLP-1 RAs also have not identified predictors of patients being able to come off insulin [10–12]. Babenko et al. showed that higher HbA1C and GLP-1 levels at baseline predicted a better glycemic response to GLP-1 RA therapy [10]. A retrospective observational study to identify predictors of response to exenatide showed that only higher baseline HbA1C predicted glycemic responsiveness but not patient age, gender, duration of diabetes, concomitant medications, weight or BMI [11]. When exenatide was added to optimized basal insulin, improvements in glycemic control and weight loss were observed regardless of baseline A1C, diabetes duration or BMI [12].

In this study, a random C-peptide was chosen as a measure of residual endogenous insulin secretion. Random C-peptide collected the day of the clinic visit is not only more convenient than stimulated C-peptide measurements following glucagon or a mixed meal tolerance test, but studies have shown random C-peptide to be a reliable marker of endogenous insulin secretion correlating well with mixed meal C-peptide levels [22]. We hypothesized that a higher random C-peptide would be associated with a more robust response to GLP-1 RA treatment. In this study, among 49 patients who had a random C-peptide measured prior to the initiation of GLP-1 agonist therapy, a normal as opposed to low C-peptide at baseline, rendered patients more likely to successfully discontinue prandial insulin when a GLP-1 agonist was added. A higher baseline C-peptide also predicted a greater degree of HbA1C lowering, though not significantly so, likely due to smaller patient numbers. A greater percentage of patients having a normal/high C-peptide were able to discontinue insulin than those with a baseline low C-peptide in our study. This was statistically significant at 3 months.

Other studies have provided mixed evidence to support the usefulness of C-peptide determination to predict responsiveness to other noninsulin antihyperglycemic agents. Higher C-peptide values predict HbA1C lowering by thiazolidinediones [18, 19]. A study by Song et al. demonstrated that fasting and stimulated C-peptide levels were significantly associated with a reduction in HbA1C in a prospective study of 73 patients treated with exenatide not on insulin [13]. Fasting C-peptide has been shown to predict HbA1c-lowering when GLP-1 agonists are added to patients with type 2 diabetes [17]. Higher baseline fasting C-peptide predicts responsiveness to rosiglitazone [18], and normal fasting C-peptide predicts a good response to rosiglitazone [19] and DPP-4 inhibitor therapy [20, 21]. Another study showed a lack of predictive value with respect to improvement in glycemic control when C-peptide was tested prior to initiating the DPP-4 inhibitor sitagliptin [23]. A higher meal-stimulated C-peptide predicted responsiveness to treatment with the combination of metformin and glibenclamide [24]. However, fasting and glucagon-stimulated C-peptide levels did not predict responsiveness to metformin [25], nor did random C-peptide testing predict responsiveness to combination of metformin and a sulfonylurea [26] when added to insulin-treated patients with type 2 diabetes. A 60-minute test meal C-peptide level was found to successfully predict switching from insulin therapy to liraglutide monotherapy among 69 patients with type 2 diabetes who were on a low 1.9-unit average daily insulin dose [27]. Shorter diabetes duration also predicted a successful switch from insulin to liraglutide.

As this was a retrospective, nonrandomized study, there are certain factors that were not accounted for including lifestyle modification and the addition of other antihyperglycemic medications at different time points. Many study subjects followed in our diabetes center received primary and specialty care by clinicians outside of our medical system. We were unable to confirm whether concomitant conditions were present that may have impacted their baseline or follow-up diabetes control including infections, oral or injected steroids, or other medications or medical illnesses. Data for the study was captured when patients were seen for follow-up clinic visits. For this reason, there were missing HbA1c, insulin dose, and other data points affecting *N* values. There was no severe hypoglycemia observed during the interval patients were studied, and patient-reported hypoglycemia was infrequent. Continuous glucose monitor use would be useful in future studies to better capture rates of hypoglycemia among insulin-treated patients placed on GLP-1 RAs. While previous studies have suggested that female patients may be more responsive to GLP-1 RA therapy, given the sample size, the effect of sex on glycemic outcomes based on random C-peptide level was not studied. Future, larger studies addressing this question should be considered [28].

In conclusion, this study contributes to the existing body of evidence supporting the efficacy of GLP-1 RAs added to insulin therapy in patients with uncontrolled type 2 diabetes. A good glycemic response was seen following the addition of GLP-1 RA therapy with HbA1C reductions ranging from 0.40 to 0.94% regardless of baseline insulin dosage, baseline BMI, or duration of diabetes. This study confirms the value of adding GLP-1 RAs to patients even on basal-bolus insulin regimens, or on high insulin doses (the mean total daily dose at baseline was 82 units). Further, it provides guidance for the use of random C-peptide testing as a relative predictor of patients being able to discontinue prandial insulin therapy. Future larger, randomized studies will be needed to better quantify anticipated reductions in insulin requirements based on baseline patient characteristics.

## Figures and Tables

**Table 1:** Baseline demographics and clinical features.

Age at baseline, mean ± SD	61.49 ± 11.36
Sex, *n* (%)	
Male	51 (63.75)
Female	29 (36.25)
Race/ethnicity, *n* (%)	
White	44 (55)
Black	25 (31.25)
Asian	2 (2.5)
Hispanic	2 (2.5)
Other	7 (8.75)
BMI, mean ± SD (kg/m^2^)	34.42 ± 6.61
Duration of diabetes Mean ± SD (years)	16.91 ± 9.58
Years on insulin Mean ± SD (years)	7.59 ± 6.15
Random C-peptide, mean ± SD (ng/ml)	2.00 ± 1.06
Use of noninsulin agents, *n* (%)	
Metformin	54 (67.5)
Other	44 (54.32)
Choice of GLP-1, *n* (%)	
Dulaglutide	18 (22.5)
Liraglutide	51 (63.75)
Semaglutide	11 (13.75)
HbA1C, mean ± SD, %	8.23 ± 1.45
Total daily dose insulin, units	82.39 ± 72.34

**Table 2:** Change in HbA1C and change in weight at 3, 6, and 12 months from baseline among all primary analysis patients^a^.

	3 months	*p* value	6 months	*p* value	12 months	*p* value
% change in A1C (mean ± SD)	−0.94 ± 0.26	0.0007	−0.40 ± 0.21	0.0636	−0.58 ± 0.23	0.0154
Change in weight (mean ± SD in kg)	−4.90 ± 1.40	0.001	−4.41 ± 0.79	<0.0001	−5.64 ± 0.86	<0.0001

^a^For nonmissing paired data.

**Table 3:** Number of patients to discontinue prandial insulin based on random C-peptide (low <0.8 ng/ml vs. normal ≥0.8 ng/ml).

		Low C-peptide	Normal C-peptide	*p* (2-sided Fisher's exact test)
3 months	Discontinued prandial insulin	0	11	
	Total	7	23	0.029
6 months	Discontinued prandial insulin	0	8	
	Total	4	20	0.262
12 months	Discontinued prandial insulin	1	7	
	Total	8	23	0.642

**Table 4:** Change in A1C in low C-peptide group vs. normal C-peptide group at each time point.

	*N*	Low C-peptide	Normal C-peptide	*p* value (*t* test)
		9	40	
3 months	*n*	3	21	
	Mean (s.e.)	-0.633 (0.120)	-1.014 (0.300)	0.252
	SD	0.208	1.376	
	Median (IQR)	-0.700 (-0.800, -0.400)	-1.000 (-1.900, -0.300)	
	Missing	6	19	
6 months	n	5	21	
	Mean (s.e.)	-0.720 (0.156)	-0.667 (0.259)	0.863
	SD	0.349	1.188	
	Median (IQR)	-0.900 (-0.900, -0.400)	-0.700 (-1.300, 0.100)	
	Missing	4	19	
12 months	*n*	9	23	
	Mean (s.e.)	-0.678 (0.255)	-0.913 (0.278)	0.539
	SD	0.766	1.335	
	Median (IQR)	-0.300 (-1.000, -0.200)	-0.900 (-1.700, -0.200)	
	Missing	0	17	

**Table 5:** Mean insulin total daily dose reduction in patients who discontinued vs. remained on prandial insulin at each time point^a^.

Patient population	3 months		6 months		12 months	
	Mean TDD^b^ reduction	SD	Mean TDD reduction	SD	Mean TDD reduction	SD
Discontinued prandial insulin	49.42	48.59	84.38	70.29	52	18.02
Remained on prandial insulin	19.43	31.7	24.5	46.56	30.91	58.11

^a^Includes all primary analysis patients who were on prandial insulin at baseline. ^b^Total daily dose (in units).

## Data Availability

Data is available on request.

## Additional Points

*Highlights*. Physicians lack clear guidance on which patients will respond most robustly to GLP1 receptor agonists and what insulin adjustments should be made when these medications are added. We found in a small cohort that a normal-to-high C-peptide may be a useful predictor of patients able to discontinue or significantly reduce their insulin doses when a GLP-1 receptor agonist is added. Other baseline patient characteristics such as BMI, diabetes duration, and total daily dose of insulin, do not appear to clearly predict response.
